# Sellers’ Revisited: A Big Data Reassessment of Historical Outbreaks of Bluetongue and African Horse Sickness due to the Long-Distance Wind Dispersion of *Culicoides* Midges

**DOI:** 10.3389/fvets.2017.00098

**Published:** 2017-07-20

**Authors:** Peter A. Durr, Kerryne Graham, Rieks D. van Klinken

**Affiliations:** ^1^CSIRO Australian Animal Health Laboratory, East Geelong, VIC, Australia; ^2^CSIRO Health and Biosecurity, Dutton Park, QLD, Australia

**Keywords:** aerial dispersion, African horse sickness, big data, bluetongue, cloud computing, *Culicoides*, *TAPPAS*

## Abstract

The possibility that outbreaks of bluetongue (BT) and African horse sickness (AHS) might occur *via* long-distance wind dispersion (LDWD) of their insect vector (*Culicoides* spp.) was proposed by R. F. Sellers in a series of papers published between 1977 and 1991. These investigated the role of LDWD by means of visual examination of the wind direction of synoptic weather charts. Based on the hypothesis that simple wind direction analysis, which does not allow for wind speed, might have led to spurious conclusions, we reanalyzed six of the outbreak scenarios described in Sellers’ papers. For this reanalysis, we used a custom-built Big Data application (“*TAPPAS*”) which couples a user-friendly web-interface with an established atmospheric dispersal model (“*HYSPLIT*”), thus enabling more sophisticated modeling than was possible when Sellers undertook his analyzes. For the two AHS outbreaks, there was strong support from our reanalysis of the role of LDWD for that in Spain (1966), and to a lesser degree, for the outbreak in Cyprus (1960). However, for the BT outbreaks, the reassessments were more complex, and for one of these (western Turkey, 1977) we could discount LDWD as the means of direct introduction of the virus. By contrast, while the outbreak in Cyprus (1977) showed LDWD was a possible means of introduction, there is an apparent inconsistency in that the outbreaks were localized while the dispersion events covered much of the island. For Portugal (1956), LDWD from Morocco on the dates suggested by Sellers is very unlikely to have been the pathway for introduction, and for the detection of serotype 2 in Florida (1982), LDWD from Cuba would require an assumption of a lengthy survival time of the midges in the air column. Except for western Turkey, the BT reanalyses show the limitation of LDWD modeling when used by itself, and indicates the need to integrate susceptible host population distribution (and other covariate) data into the modeling process. A further refinement, which will become increasingly important to assess LDWD, will be the use of virus and vector genome sequence data collected from potential source and the incursion sites.

## Introduction

Bluetongue (BT) and African horse sickness (AHS) are major animal diseases affecting mostly sheep and horses, respectively (1, 2). Although the actual disease syndromes are distinct, they share much in common including a high mortality rate in susceptible animals and being transmitted by blood-feeding *Culicoides* spp. (family *Ceratopogonidae*). Furthermore, both diseases are caused by segmented viruses of the same genus (*Orbivirus*) and are reportable (“notifiable”) to the OIE. Currently, BT is a serious re-emerging disease, having caused a number of major epidemics in Europe since 1998 (3, 4). AHS has been less of a problem in recent years due to the development of an effective vaccine; nevertheless, it remains the major transboundary animal diseases of equines, and thus causes restrictions on their movements within and from endemically infected countries (5).

Due to the seriousness of both diseases, recent outbreaks have generally been well investigated, and the data and learnings have informed subsequent risk assessments. From these, the risk pathways of introduction are now generally well described, and increasingly quantified (5–8). A major risk pathway for both diseases that has been consistently identified is the movement of viremic animals, and for example, the last major outbreak of AHS in Europe (between 1987 and 1990) is considered to have been caused by the import from Namibia of (presumably) viremic zebras to a safari park in central Spain (9). Nevertheless, determining the exact pathway of introduction of the two diseases is often problematic, as judged by the extensive investigations into the possible source of the BTV-8 serotype which was introduced into the Netherlands in 2006 (10). These investigations considered all potential mechanisms of introduction, but were only able to conclude that “the exact origin and route of the introduction of BTV-8 thus far remains unknown” (10).

One transport route that was intensively investigated during the BTV-8 outbreaks in Northern Europe was the role of long-distance wind dispersion (LDWD) of infected *Culicoides* midges. That wind dispersion of infected midges may be a source of infection was first posited by R. F. Sellers in a paper investigating historical outbreaks of AHS in Europe, the Middle East, and India between 1943 and 1966 (11). This was followed up by comparable analyses of outbreaks of BT in Portugal (12), Cyprus (13), Turkey (14), and North America (15, 16).

The basic methodology adopted by Sellers in his investigations was to carefully review the epidemiology of the outbreaks to rule out the possibility of the movement of animals or the transport of the vector in aircraft or ships as a plausible source of introduction of the causative virus. Once this was shown, then the possibility of introduction by wind-borne *Culicoides* was assessed by determining the time window when this might have occurred, given the adjustments needed for the incubation period of the disease in the host and any delays in reporting and diagnosing the outbreak. Synoptic weather charts were then visually inspected to assess if the wind direction from a known source of infection—such as an outbreak in a surrounding country—made wind-borne dispersion possible. Based on this methodology, Sellers concluded that LDWD was possible for many of the BT and AHS incursions investigated (Table 1).

**Table 1:** African Horse Sickness (AHS) and bluetongue (BT) outbreaks between 1943 and 1988 investigated by Sellers and his collaborators for the possible role of long-distance wind dispersion of infected *Culicoides* midges as the pathway of introduction of the virus.

Disease	Outbreak Country (region)	Year of outbreak	Purported source country or region	Publication
AHS	Cape Verde Islands	1943	Senegal	(11)
**BT**	**Portugal**	**1956**	**Morocco**	(12)
AHS	India	1960	Pakistan	(11)
AHS	Iraq, Turkey, and Syria	1960	Iran	(11)
**AHS**	**Cyprus**	**1960**	**Turkey**	(11)
AHS	Algeria	1965	Sub-Saharan Africa	(11)
**AHS**	**Spain**	**1966**	**Morocco**	(11)
**BT**	**Cyprus**	**1977**	**Syria and/or Turkey**	(13)
**BT**	**Turkey (Aydin Province)**	**1977**	**Cyprus**	(14)
**BT**	**USA (Florida)**	**1982**	**Cuba**	(15)
BT	Canada (British Columbia)	1987	USA (Washington state)	(16)
BT	Canada (British Columbia)	1988	USA (Washington state)	(16)

*Outbreaks reanalyzed in this study are highlighted in bold*.

While examining wind direction is useful to determine if LDWD is a *possible* route of introduction of bluetongue virus (BTV) or African horse sickness virus (AHSV), it does not show that it is *plausible*, as there is no assessment of wind speed and the conditions affecting survival of the midges along the transport path. Thus slow-blowing winds might not enable the transport of the midges across the distance necessary for transboundary spread of the viruses, and similarly, extreme conditions of temperature or dryness might result in a high mortality of the midges (17).

At the time Sellers did most of his investigations, wind direction analysis was the only meteorological tool available, and thus these other factors could not be taken into account. However, in the past 10 years there have been considerable advances in the use of “atmospheric dispersion models” (ADM) linked to “numerical weather prediction” (NWP) reconstructions of the atmospheric conditions at the time of the dispersion (18). These advanced models were very successful in predicting the incursion of BTV-8 into eastern England (19, 20) and into southern Sweden (21), and at least for the UK, have been operationalized as an integrated “early warning system” by their national meteorological service, the Met Office (22).

As part of the validation process for the UK’s prediction system for *Culicoides*, several case studies were investigated (22). Most of these were for the outbreak of BTV-8 in northern Europe during 2007–2009, but one was based on an outbreak of BTV-2 in the Balearic Islands in the western Mediterranean during 2000. The potential for LDWD as a means of introduction of the virus to these islands had already been investigated using a back-trajectory analysis, and from this it was concluded that LDWD from Sardinia was possible (23). However, the subsequent analysis using the UK forecast system and a much greater number of back-trajectories, confirmed that while LDWD was the probable route of introduction, the most plausible source was actually north Africa (22).

This Balearic Island example clearly demonstrates the risk of bias from only choosing a limited number of trajectories and the need to explore more of the LDWD “scenario space.” This is particularly relevant where source and destination areas for LDWD are large and/or the disease or virus detection temporal windows are extended. Nevertheless, doing large numbers of LDWD runs in a timely manner is only feasible using a systems approach, as implemented by the UK Met Office (“*NAME*”), with runs undertaken in an integrated high performance computing (HPC) environment and automated spatiotemporal map outputs (22). Such a HPC-enabled application handling masses of input data is one example of what is increasingly been referred to as “Big Data.” Here, we describe the development of a Big Data system (“*TAPPAS*”) comparable to that developed by the UK Met Office’s *NAME* system, and we make use of it to retrospectively analyze six of the LDWD outbreaks investigated by Sellers.

## Materials and Methods

### Spatiotemporal Data of the BT/AHS Outbreaks Investigated by Sellers

In total, Sellers described 12 outbreaks (or detections) of BT and AHS covering a 45-year period, from 1943 to 1988 (Table 1). Of these, we reanalyzed six, being selected as those for which dispersion occurred over international borders and also over the sea. The latter was on account of unrecorded movements of animals *via* shipping being less likely than unrecorded movements over land. The outbreak of AHS in the Cape Verde Islands in 1943 satisfied this criteria, but was before the earliest date for which climate data were available for reanalysis.

From each of these publications, and also from the primary papers which described the outbreaks, we extracted the following data (Table 2).

- The location(s) where the outbreaks of BT or AHS were first detected. Latitudes and longitudes (in decimal degrees) were assigned using either the coordinates provided by Sellers or the primary publications describing the outbreaks or else through the use of online gazetteers.
- The presumed source locations of the infected midges. These varied from quite vague regions (“northern Cuba” for the BT detection of serotype 2 in Florida, “BT-USA-82”) to more exact locations (the province of Icel in Turkey for the AHS outbreak in Cyprus in 1960, “AHS-CYP-60”).
- The date the outbreak was presumed to have begun. In most cases this corresponds to when the index case was detected in the infected flock (sheep) or stable (horses). An exception is the BT outbreak in Portugal in 1956 (“BT-PRT-56”), for which the outbreak investigation undertaken by the veterinary authorities concluded that it had begun 10 days previously (24). The detection of BT serotype 2 in the USA in 1982 (“BT-USA-82”) occurred in an asymptomatic sentinel cattle herd, and thus the “outbreak” corresponds to the date viremia was first detected.
- The “transport window” when LDWD of midges might have occurred. The general method Sellers used to calculate this was by working backwards from the outbreak date and assuming a minimum and maximum incubation period in the host, and whether an infectious or an incubating midge was transported. Thus for BT-PRT-56, the incubation period range in sheep was assumed to be between 6–10 days, with an incubation period in an infected midge of 7 days. If an infectious midge was transported and the shorter incubation period in the host was assumed, the latest date transport might have occurred was the 24^th^ June, while if a newly infected midge was transported and the longer host incubation period is assumed, then transport could have occurred as early as the 13th June. For BT-USA-82 the earlier date of the transport window was based on the maximum time needed for seroconversion (18 days), and the latest possible date was determined to be the shortest time period viremia might occur following infection (2 days).
- The date or dates within the LDWD transport window when Sellers judged—following examination of the synoptic weather charts (or else the back trajectory in the case of BT-USA-82)—when transport of infected *Culicoides* was most likely.

**Table 2:** Epidemiological parameters used for the reanalyzed bluetongue (BT) and african horse sickness (AHS) outbreaks.

Outbreak	Location where the index case was detected	Potential source locations of infected *Culicoides* midges	Date the outbreak began	Long-distance wind dispersion (LDWD) transport window assigned by Sellers	Dates determined when LDWD was possible	Primary publications describing the outbreaks
BT-PRT-56	Lower Alentejo: Alcácer do Sal (38.37 N, 8.51 W)	Northern Morocco	1st July 1956	13th June 1956 to 24th June 1956	21st June 1956	(24, 31–34)
AHS-CYP-60	Famagusta: Arnadhi (35.24 N, 33.86 E)	Turkey: Icel Province (now renamed as Mersin)	6th September 1960	21st August 1960 to 1st September 1960	28th August 1960	(35, 36)
AHS-ESP-66	Province of Cardiz: Los Barrios (36.18 N, 5.48 W)	Northern Morocco (Provinces of Nador, Al Hoceima, Tetouan and Tangier)	13th October 1966	27th September 1966 to 8th October 1966	3rd October 1966	(37–40)
BT-CYP-77	Kyrenia district: Ayios Amvrosios (35.33 N, 33.58 E), Lapithos (35.34 N, 33.16 E), Karavas (35.34 N, 33.21 E); Famagusta district: Frenaros (35.04 N, 33.92 E), Vrysoules (35.09 N, 33.88 E)	Eastern Turkey, Northern Syria	20th August 1977	4th August 1977 to 15th August 1977	11th August 1977 to 14th August 1977	(43, 44)
BT-TUR-77	Aydin Province: 2 villages near the city of Aydin (37.84 N, 27.84 E)	Cyprus	24th October 1977	5th October 1977 to 19th October 1977	14th October 1977 (evening)	(46, 47)
BT-USA-82	Florida: Ona (27.43 N, 81.92 W)	Northern Cuba	OnaA: 2nd September 1982, OnaB: 8th October 1982	OnaA: 15th August 1982 to 31st August 1982, Ona B: 20th September 1982 to 6th October 1982	OnaA: 18th August 1982 (evening), OnaB: 21st to 23rd September 1982	(50, 51)

*Most of the data was extracted from the publications describing the original LDWD analyses undertaken by Sellers (see Table 1), but some were derived from the primary publications describing the outbreaks*.

### LDWD Simulation Modeling: The *TAPPAS-HYSPLIT* Application

*TAPPAS* (“Tool for Assessing Pest and Pathogen Airborne Spread”) is an integrated web-based application designed with the purpose of providing a validated system for undertaking LDWD of veterinary, medical, and agricultural pests and pathogens, particularly insects and fungal spores.^1^ The application provides a password-protected workspace where users can set up forward and backward LDWD runs, specifying the spatial extent of the source or destination and the temporal window for which the run is needed. Users are also given the option of selecting species templates whereby the biological properties of the pest/pathogen which might affect dispersion—such as take-off height and maximum survival time in the air column—can be specified.

*TAPPAS* is designed to flexibly use a number of ADM programs, as reflects the multitude which have been developed over the past 20 years to model the dispersion of aerosols, dust, and pollutants under different environmental conditions (25). In addition, the option is provided to use different NWP datasets, which vary in their spatial and vertical (“sigma level”) resolution. Currently, only one ADM program has been implemented, for which we chose *HYSPLIT* (26), on account of it being the program we previously used to assess the potential spread of *Culicoides* from Timor Leste, Indonesia, and Papua New Guinea into northern Australia (27, 28). The *HYSPLIT* FTP site lists a number of publicly available meteorological NWP datasets, for which *TAPPAS* provides user access to the NCEP–NCAR global reanalysis dataset (1948–present; 2.5° horizontal resolution) and the NCEP/North American Mesoscale dataset (2007–present; 12 km horizontal resolution). In addition, *TAPPAS* can use the Australian Bureau of Meteorology (“BOM”) *ACCESS* global dataset (2014–present, ~40 km horizontal resolution) (29).

**Figure 1:**
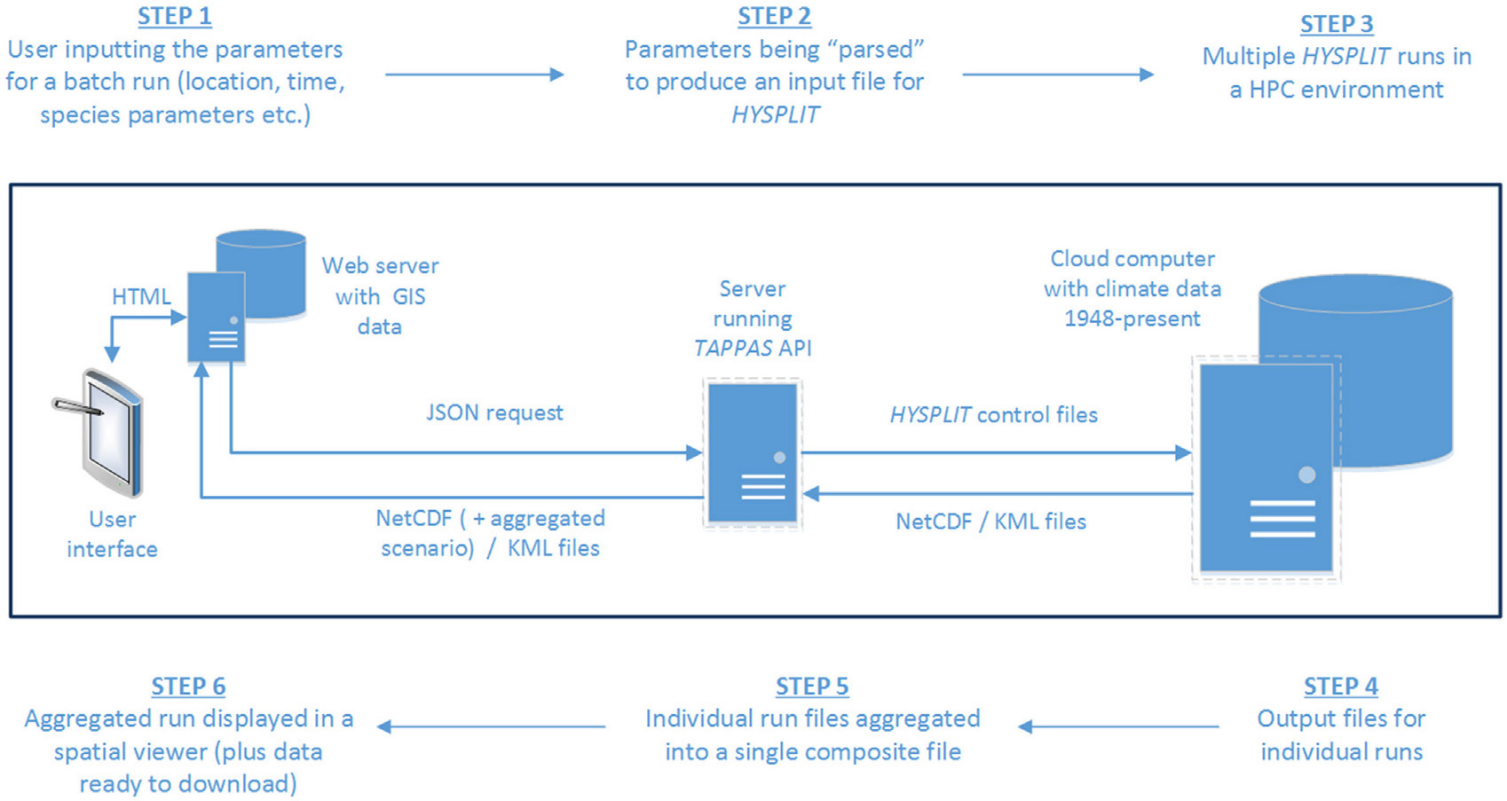
A schematic of the data-flows for the setting up of a *TAPPAS* batch run, starting from the user inputting the required parameters (step 1) and finishing with them receiving back on-screen maps of the results of the simulation, with the option to download the data for further post-processing (step 6).

The process of setting up a *TAPPAS* batch run leads to a “request” (in JSON format) which contains all the parameters needed to undertake the specified number of individual *HYSPLIT* runs (Figure 1). This JSON request is constructed on the web-server running *TAPPAS* which then delivers it to a custom-built web-based Application Programming Interface (API) running on a cloud computer within a supercomputer infrastructure. This API acts as a “gatekeeper” to access the supercomputing infrastructure, ensuring that the JSON request contains all the required parameters and poses a minimized IT security threat. The JSON request is then parsed by the API to produce *HYSPLIT* control files, with one for each of the individual dispersion runs specified during the setup of the *TAPPAS* scenario. *HYSPLIT* then sequentially undertakes each of the runs, using the climate dataset specified in the set-up, and thereafter producing its standard output files. The API then aggregates these and delivers them back to the web-server, where the user can view the run output.

**Figure 2:**
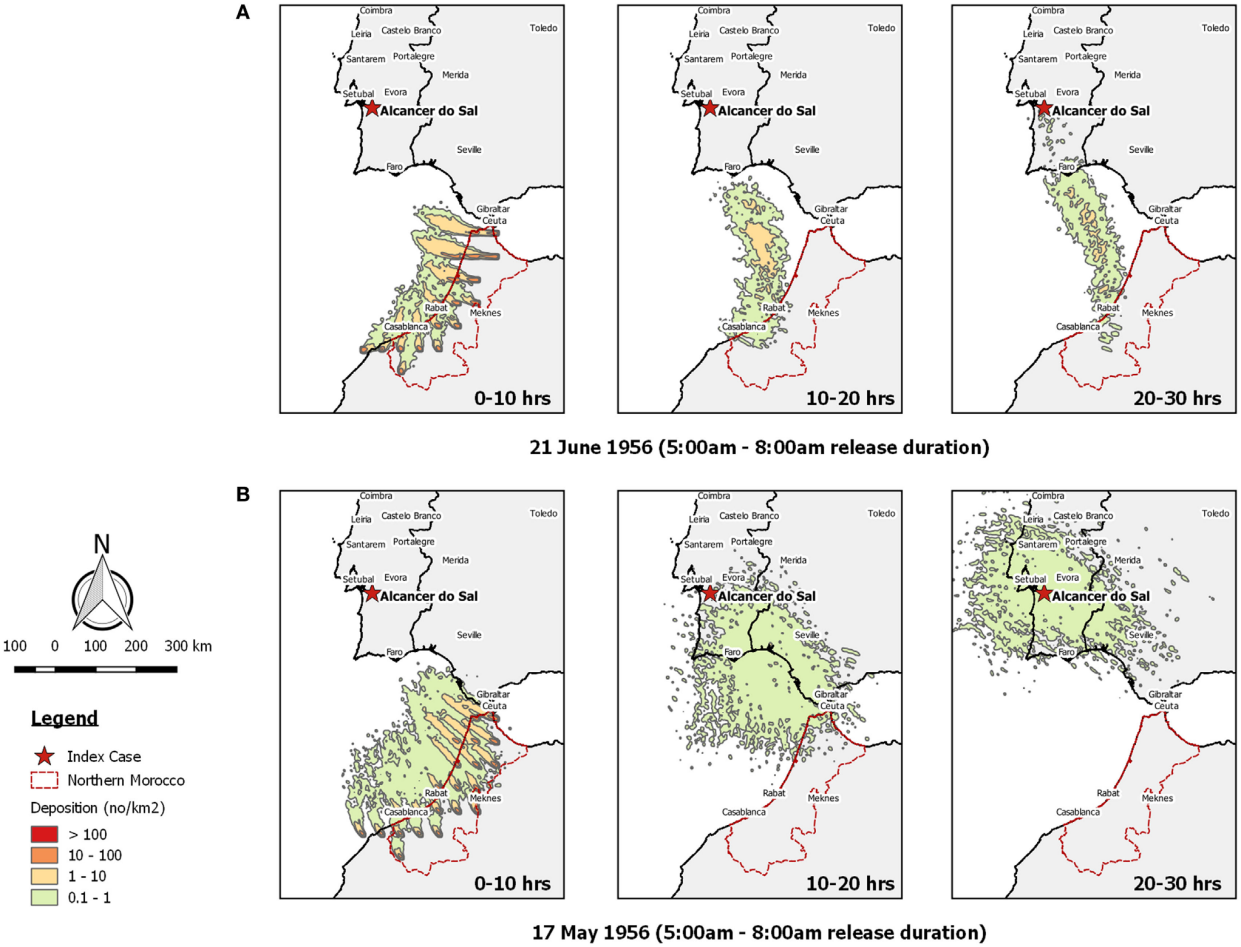
Post-processed *TAPPAS* output for BT-PRT-56 showing forward dispersion from Morocco for three transport durations (0–10, 10–20, and 20–30 h) for morning releases of 10,000 particles/h/site on **(A)** the 21st June 1956 and **(B)** the 17th May 1956.

Two standardized visual outputs are provided in a spatial viewer which uses *OpenLayers*.^2^ The first follows the run from the selected source point(s) for the duration specified during the set-up phase. This allows users to visually examine how the concentration of particles dynamically are transported and deposited (e.g., Figure 2). An alternative output is provided which accumulates the deposition over the course of the run (e.g., Figure 6). Both of these visualizations are available for “forward” runs, when a user wants to investigate where and how many particles will be deposited from source locations, and also for “backwards” runs, when a user would like to determine possible source locations given a specific outbreak location (e.g., Figure 8).

The spatial viewer is useful to enable users to interactively explore the data, but the screen resolution is insufficient for publication-quality images. *TAPPAS* thus provides the option of downloading the NetCDF dataset for further post-processing or else KML files for viewing and manipulating within a geographic information system.

### *TAPPAS* Run Parameters and Epidemiological Interpretation of the Results

For the reanalysis runs, we developed a *TAPPAS* species template for *Culicoides*, which defines those parameters which enable the passive dispersion of small insects to be represented as *HYSPLIT* particles (Table 3). The parameter values were based on previously published studies which used *HYSPLIT* to investigate LDWD of *Culicoides* from Timor Leste, Indonesia, and Papua New Guinea into northern Australia (27). As *HYSPLIT* has some redundancy in its parameterization, not all of these need to be specified, and instead are given default values. For example the diameter and density of the particles are given default of values of “1.0” if the velocity for dry deposition is provided.

**Table 3:** The *TAPPAS* template biophysical parameter values used for the reanalysis of Sellers’ presumed long-distance wind dispersion outbreaks.

Template parameter	Value assigned for the Sellers’ reanalyses	Explanatory notes
Release quantity	10,000/h	Based on a mid-range value of those examined by Eagles *et al*. (27)
Release duration	3 h	Total release over a 3-h period is a maximum of 30,000 particles
Release height	1 m above ground level (AGL)	Based on average height of sheep, cattle, and horses (at withers)
Top of model	2,000 m AGL	Based on a mid-range value of those examined by Eagles *et al*. (27)
Model run time (from first release)	30 h	Duration of the model run set at 30 h to equate with the maximum survival time (see below)
Max. time in air column (alive and infective)	30 h	Assumes maximum time *Culicoides* midges survive in the air column is 30 h
Velocity (of dry deposition)	0.005 m/s	Equates with gravitational settling. Upper value of estimates provided by Agren *et al*. (21)
In cloud (wet deposition)	8 × 10^−5^ l/l	Default *HYSPLIT* value
Below cloud (wet deposition)	8 × 10^−5^ l/s	Default *HYSPLIT* value
Sampling output	10 h	Allow deposition to be visualized in ranges 0–10 h, 10–20 h, and 20–30 h
[Diameter]	1.0	Default value—but not needed as a dry deposition velocity parameter provided (see above)
[Density]	1.0 g/cc	Default value—but not needed as a dry deposition velocity parameter provided (see above)
[Lifespan]	15 days	Obtained from Mellor *et al*. (73) who stated survival was between 10 and 20 days; However, the parameter was not used for the Sellers’ reanalyses as in all cases the maximum time in air column was presumed to be much less

*Some of the template parameters (enclosed by square brackets) were not needed for the Sellers’ runs, but are provided by means of explanation*.

Using the *TAPPAS* interface, we set up batch runs for the LDWD spatiotemporal windows indicated in Table 2. For the outbreaks where more precise geographical locations had been defined by Sellers (e.g., AHS-ESP-66), we used these as a point source origin, but when these locations were less specific we selected either a broad geographical region (e.g., BT-CYP-77) or else a named province (e.g., AHS-CYP-60). When the source was an area, then the dispersion was modeled solely from the centroid of the underlying *TAPPAS* grid which was thus assumed to represent dispersion from the whole grid square (approximately 55 km × 55 km at the equator). All morning dispersions were started from 05:00 local time, with a gradual release over a 3-h period. Similarly, all evening dispersions commenced at 17:00 local time with a 3-h gradual release. Thus for a grid-based run each individual morning or evening run represented approximately a total of 10 uplifted particles per square kilometer.

Where runs showed that LDWD was unlikely to have occurred in the spatiotemporal window defined by Sellers, we extended the modeling backwards in time, to test the hypothesis that introduction of the virus might have occurred sometime before the detection. For some runs, we also extended the duration of transport and the number of uplifted midges (Table 3). Although for exploration of the output of the runs we used the inbuilt *TAPPAS* map viewer, for the final output shown here we downloaded the KML files and post-processed the data using *QGIS* 2.14.3 Essen. *HYSPLIT* by default calculates its deposition output as the number of particles per square meter, but to make this output more biologically meaningful, we converted these values to a density per square kilometer.

Although Sellers did not use the term “risk assessment” at the time of publishing his research, his work can be re-interpreted as fitting within this paradigm, *viz*., to assess the risk of introduction of BT or AHS *via* infected midges being carried on the wind. However, unlike more recent risk assessments that have been carried out to answer this question (20), the investigations undertaken by Sellers did not attempt to estimate the likelihood of infected midges being uplifted in the wind, and no data were provided on either the size of the host population or that of the midges at the source. This applies equally to our reanalyses, and thus in reporting our results we have avoided terms which imply quantification of risk (“high risk,” “low risk,” etc.) and instead have used more subjective terms (“possible,” “plausible”) to reassess whether the outbreaks described by Sellers might have been caused by LDWD (Table 4).

**Table 4:** Terminology used for our reanalyses to conclude whether long-distance wind dispersion might have been responsible for the outbreaks of bluetongue and African horse sickness investigated by Sellers.

Risk-associated term	Spatial extent of deposited particles	Transport time	Number of deposited particles at outbreak destination
Not possible	Dispersion plume does not intersect with the locations in which the outbreak occurred	Transport time > 30 h	No particles deposited
Possible but less likely (scenario 1)	Dispersion plume covers the locations in which the outbreak occurred	Transport time > 20 h (but less than 30 h)	≥1 particle/km^2^
Possible but less likely (scenario 2)	Dispersion plume covers the locations in which the outbreak occurred	Transport time <20 h	<1 particle/km^2^
Possible and likely (“plausible”)	Dispersion plume covers the locations in which the outbreak occurred	Transport time <20 h	≥1 particle/km^2^

## Results

### BT-PRT-56

The 1956 outbreak of BT in southern Portugal and southwest Spain, subsequently determined to be caused by BTV serotype 10 (30), was the first recognition of the disease in Europe. Because of the extensive losses among the sheep population the disease was given prominent attention by the veterinary authorities (31, 32). Once BT was confirmed, a vaccination campaign was initiated, which quickly bought the disease under control (24). At the time, epidemiological investigations on how the virus was introduced were inconclusive. There were no records of importation of wild or domestic ruminants immediately before the outbreak, and the only conclusion was that it might have been *via* the transport of *Culicoides* in aircraft or on ships (32). However, Sellers considered both these routes unlikely as such an introduction would have resulted in outbreaks near Lisbon or in central Portugal. Instead, he reasoned that Morocco might have been the source of the virus, as outbreaks were reported there in October 1956 (33, 34), and he presumed that the disease had already been in the country at least from June of that year.

Examining the *TAPPAS* dispersion run for the 21st of June 1956—the date Sellers judged when LDWD had occurred—confirmed that during this time, the wind was blowing in the direction from Morocco to southwest Portugal (Figure 2A). However, this also showed that the velocity of the wind was insufficient for infected *Culicoides* midges to have reached Portugal within the 10-h flight assumed by Sellers. Similarly extending the flight duration to 20 h would not enable the midges to make landfall, but with a 30-h flight duration, a few infected midges might have reached southern Portugal.

On the basis of it being very unlikely that such a small number of infected *Culicoides* could survive a 20- to 30-h flight and then initiate an outbreak, we extended the window of possible dispersion to the previous 6 weeks. During this time, we found one morning when a possible LDWD event might have occurred, on the 17th May 1956 within a 10- to 20-h transport time (Figure 2B). However, the density of midges reaching Portugal was low (<1/km^2^), and on the basis of our risk ranking (Table 4), we would classify this LDWD event as being possible, but not a very likely source of the 1956 BT outbreak.

### AHS-CYP-60

An epidemic of AHS started in Iran in March 1960 and quickly spread to eastern Turkey and Iraq in May and was present in southern Turkey from July onward (11, 35). On the 6th September, an outbreak was recorded in eastern Cyprus, in the Famagusta district (Table 2). This was despite the Cypriot veterinary authorities having taken strict precautions to limit the introduction of the virus, including spraying insecticide within incoming aircraft (36). Illicit entry of infected horses was considered unlikely due to heightened surveillance, and the fact that at that time, Cyprus was a large exporter, and not an importer, of mules and donkeys.

**Figure 3:**
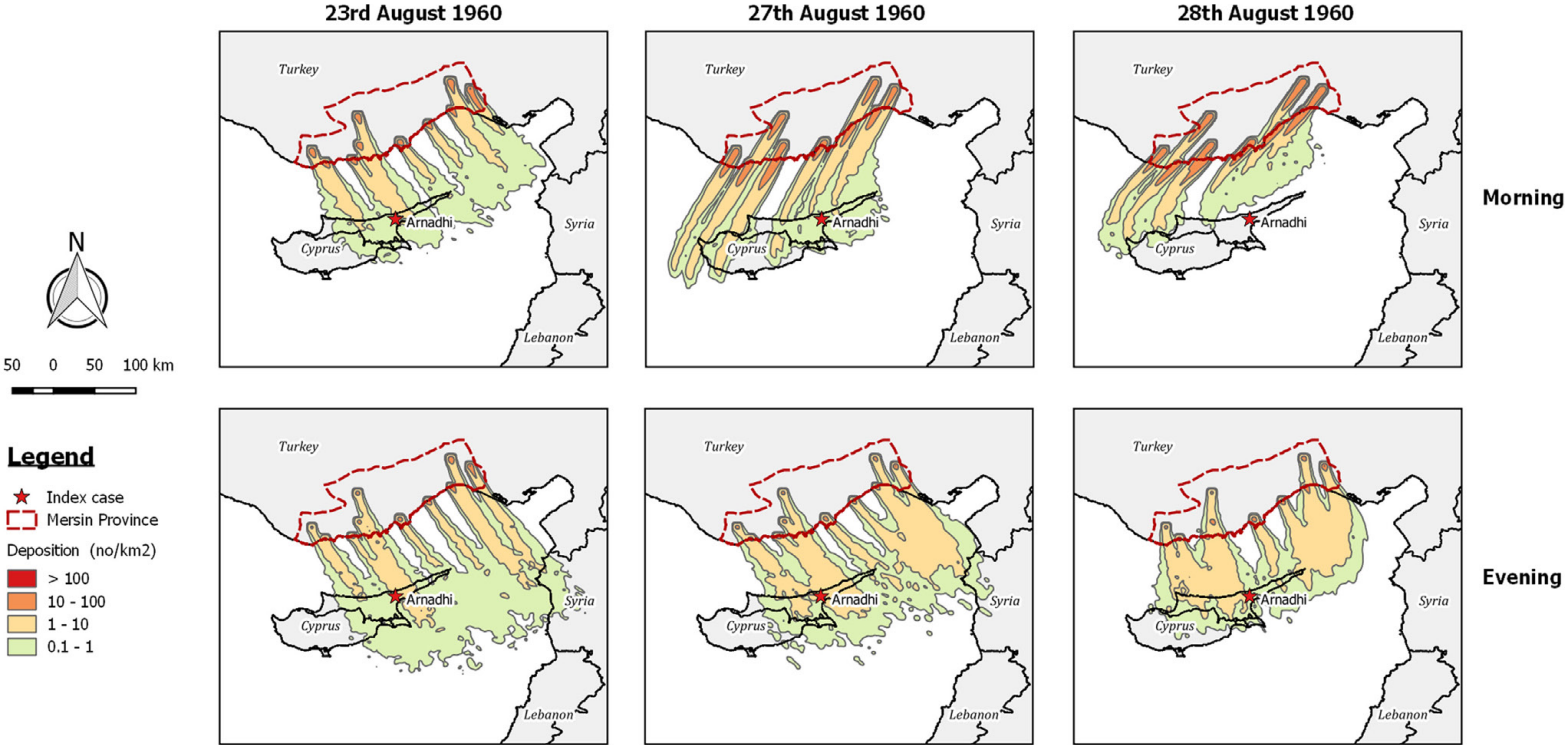
Post-processed *TAPPAS* output for AHS-CYP-60 showing forward dispersion from Mersin Province in Turkey for 0–10 h with the release of 10,000 particles/h/site for the morning and evening for 3 days (23rd August, 27th August, and 28th August 1960) where deposition occurred over Arnadhi, which was the index case site for the African horse sickness (AHS) outbreak in Cyprus.

After a careful examination of the meteorological conditions during the weeks preceding the outbreak, Sellers concluded that during the period 23rd–29th August, dispersion of infected midges from the province of Icel (Mersin) in southern Turkey to Cyprus was possible, and that infection was most likely introduced on the 28th August. Examining the dispersion output produced by *TAPPAS* for this date shows that LDWD of midges from Mersin to Cyprus was indeed possible, although only for the evening (Figure 3). Extending the *TAPPAS* runs for the incursion window identified other days when LDWD of infected midges was possible, and on this basis we concur with Sellers that that this was a plausible incursion pathway for the introduction of AHS into Cyprus.

### AHS-ESP-66

In February 1966 an outbreak of AHS (serotype 9) was detected in the southwest of Morocco, in the then province of Agadir (37, 38). The disease spread along the coast, reaching the northernmost provinces in late August. Between the 25 and 30 September, it was at its peak in the Tangier province (39), which faces Spain across the Strait of Gibraltar. Fully aware of the threat, the Spanish veterinary authorities took strict precautions, banning the entry of live animals and disinfecting and treating trucks arriving from Africa with insecticide (40). Nevertheless, between the 13th and 16th October, several outbreaks occurred on farms just inland from the coastal towns and ports in the province of Cardiz. The veterinary authorities acted with determination, and the outbreak was soon bought under control.

**Figure 4:**
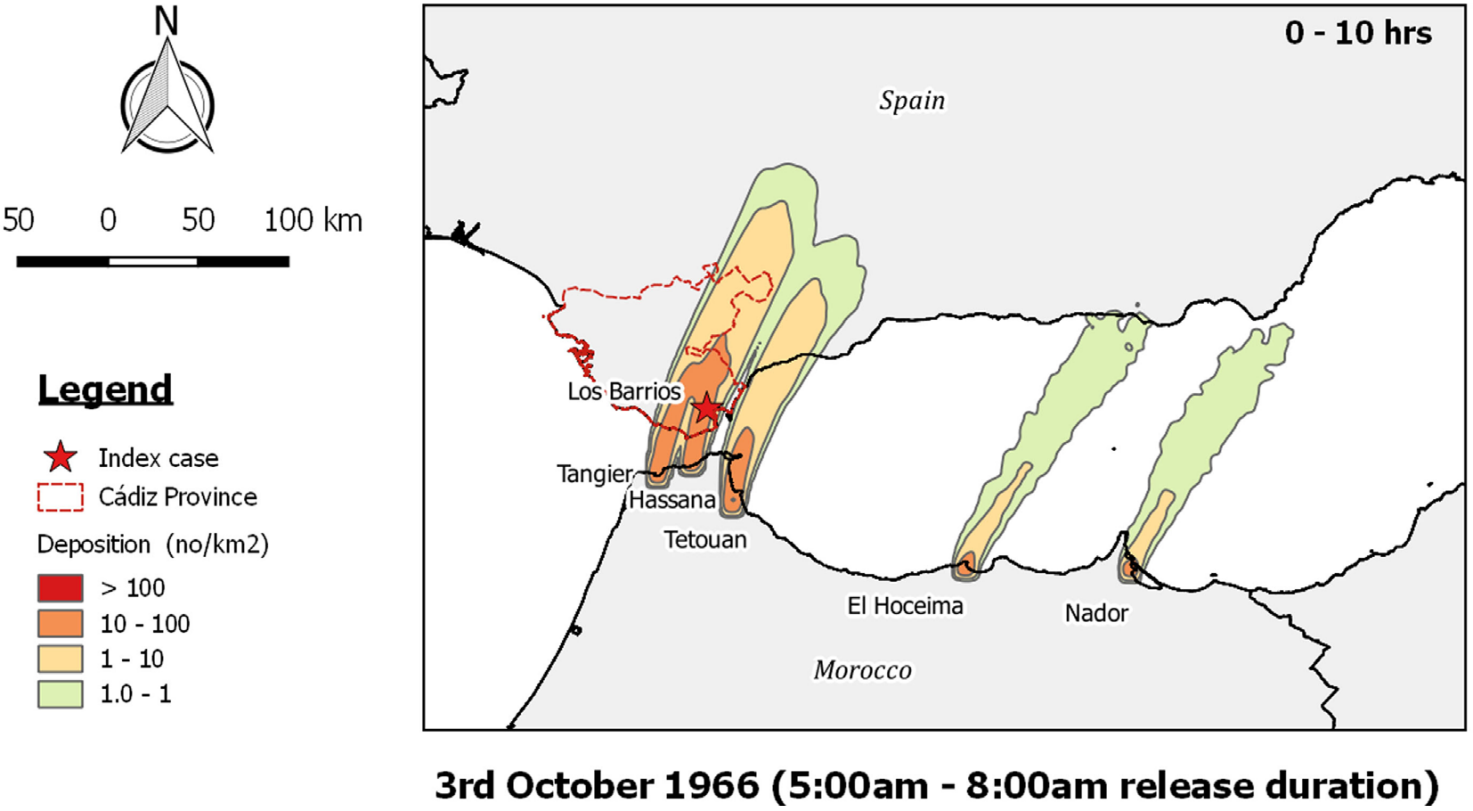
Post-processed *TAPPAS* output for AHS-ESP-66 showing forward dispersion from the outbreak locations on the north coast of Morocco on the morning of the 3rd October 1966 for a transport duration of 0–10 h with the release of 10,000 particles/h/site.

Owing to the existence of a weather station at the nearby territory of Gibraltar, Sellers was able to determine wind direction quite accurately. Throughout the possible time period of infection (27th September to 8th October) the winds were mostly from the west to southwest and east to northeast, except for 1 day, on the 3rd October, when the winds were from the south to southwest. This was accordingly the day that Sellers considered when the infected midges might have been transported into Spain. This is supported by the *TAPPAS* run, where the zone of maximum deposition was precisely where the initial outbreak occurred (Figure 4), and on this basis we conclude that LDWD was a very plausible pathway of introduction.

### BT-CYP-77

Bluetongue, caused by serotype 3, was first identified in Cyprus in 1943, although possibly it was first introduced into the country earlier as outbreaks of suspect BT (diagnosed at the time as “stomatitis”) intermittently occurred between 1924 and 1939 (41). The 1943 outbreak was more symptomatic of classic BT, and this led to the forwarding of samples to the reference laboratory in South Africa, where the diagnosis was confirmed (42). Outbreaks were then regularly reported until 1969, after which the country was free until August 1977. The 1977 outbreak was caused by a serotype 4 virus, and was identified almost simultaneously in two separate parts of the island, in the southeast (in the district of Famagusta) and the north coast (the district of Kyrenia) (43). It then spread to most of the island, but was successfully controlled by the end of October 1977 (44).

Sellers was well familiar with the Cyprus outbreak, as his institute, the Animal Virus Research Institute (Pirbright, England), had been undertaking integrated studies of the disease, including identifying *Culicoides* vectors for BTV and AHSV, since the 1969 outbreak (41, 45). He was thus well placed to be able to rule out other hypotheses of the origin of the epidemic, including persistence of the virus and introduction of carrier animals (13). His conclusion after examining synoptic weather charts was that the conditions existed for the transport of infected *Culicoides* midges between the 11 and 14 August, 1977, and this would account for the simultaneous outbreaks in both the north and southeast of the island.

**Figure 5:**
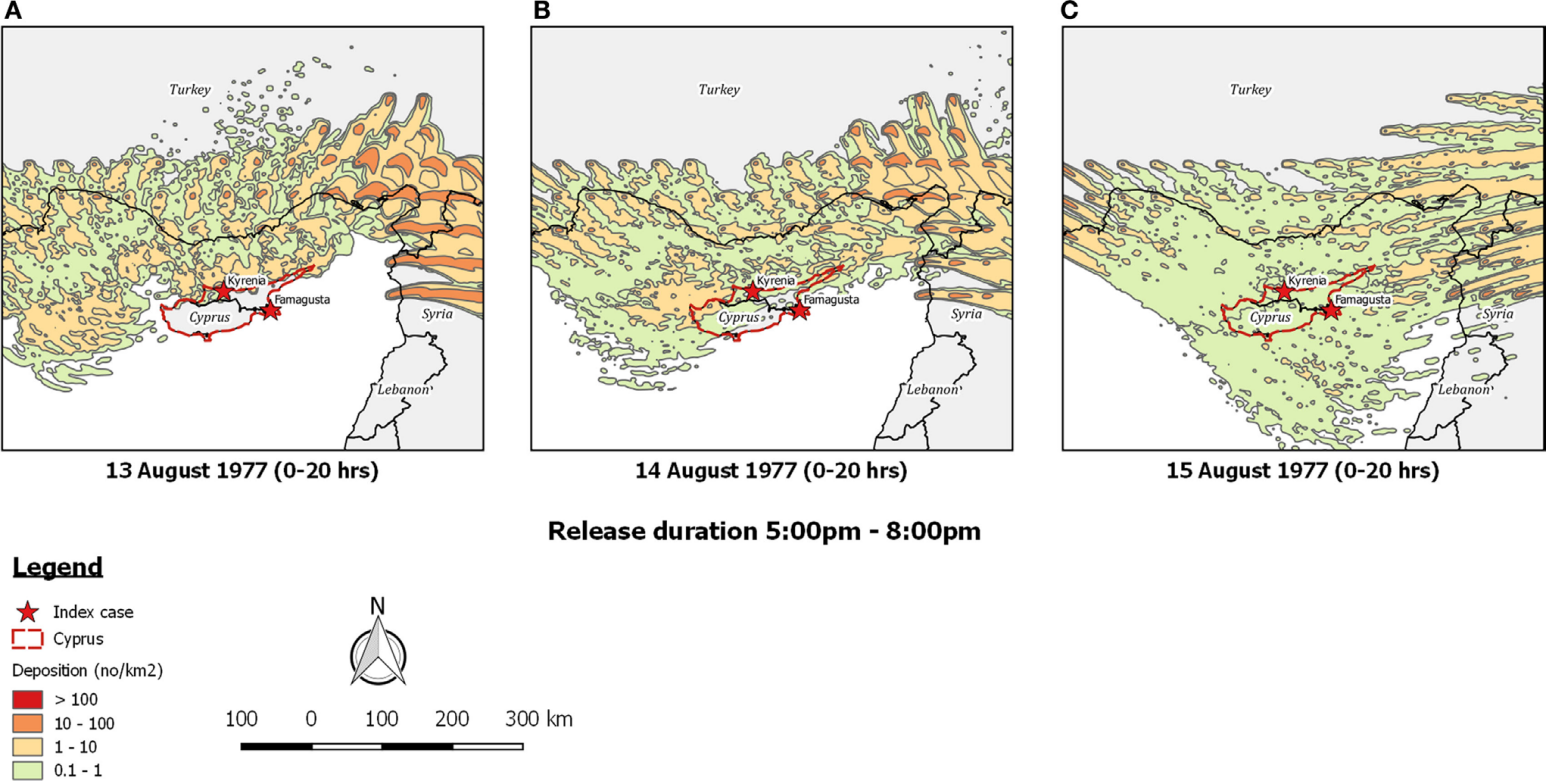
Post-processed *TAPPAS* output for BT-CYP-77 for a forward dispersion simulation from the purported source region for infected *Culicoides* midges in southern Turkey and northwestern Syria for the evenings of **(A)** 13th August 1977, **(B)** 14th August 1977, and **(C)** 15th August 1977. All transport durations were of 20 h and with the release of 10,000 particles/h/site.

**Figure 6:**
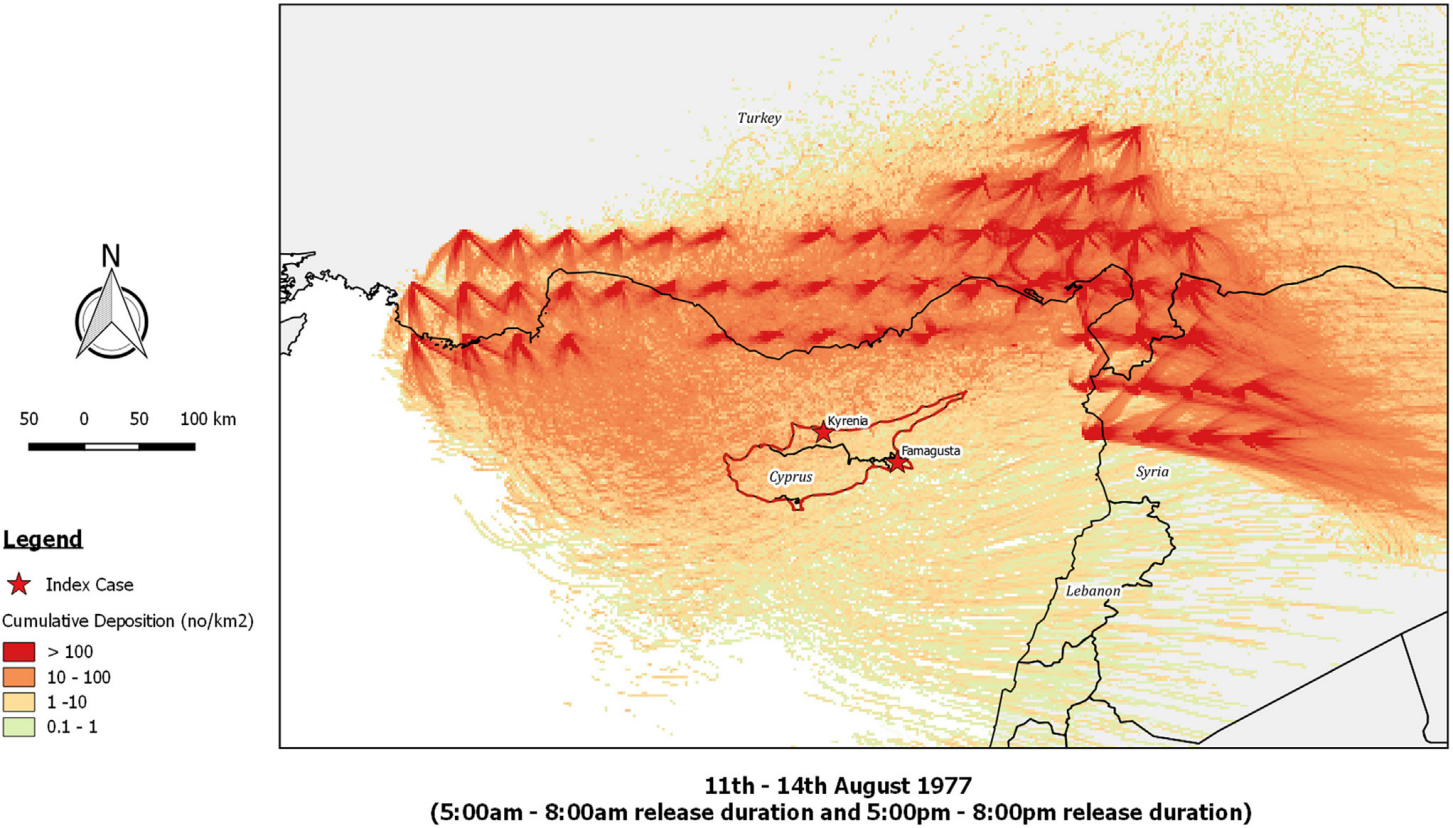
Post-processed *TAPPAS* output for BT-CYP-77 for the source region of southern Turkey and northwestern Syria for the cumulative deposition of particles following releases on the 11–14th August 1977. All transport durations were of 20 h and with the release of 10,000 particles/h/site.

Re-running the outbreak scenario using *TAPPAS* showed that during the period 11th–14th August low-density LDWD events might have occurred on the evenings of both the 13th and the 14th, and these could possibly explain the outbreaks in Kyrenia (Figures 5A,B). While neither of these dispersions would have enabled infected midges to reach the southeast of the island, implementing a *TAPPAS* run for the evening of the 15th of August showed that LDWD was possible for this outbreak, albeit at a low density (Figure 5C). However, cumulating the number of deposited particles over the period of the 11th–14th August indicates that deposition was widespread (Figure 6), although given this, it is uncertain why the two outbreaks were confined to relatively small areas of the island.

### BT-TUR-77

In October 1977 there was a severe outbreak of BT (serotype 4) in sheep in villages surrounding the city of Aydin in western Turkey (46). Previously the region had been free of BT, the last recorded outbreak having occurred in 1947. Investigations of the source of the outbreak were inconclusive, but considering that it followed soon after that in Cyprus and was of the same serotype, it was suspected that the two outbreaks were related (46). Later, and undoubtedly as a result of Sellers’ investigations of the possibility of LDWD causing the Cyprus outbreak, it was suggested that wind might have carried infected midges to western Turkey (47).

**Figure 7:**
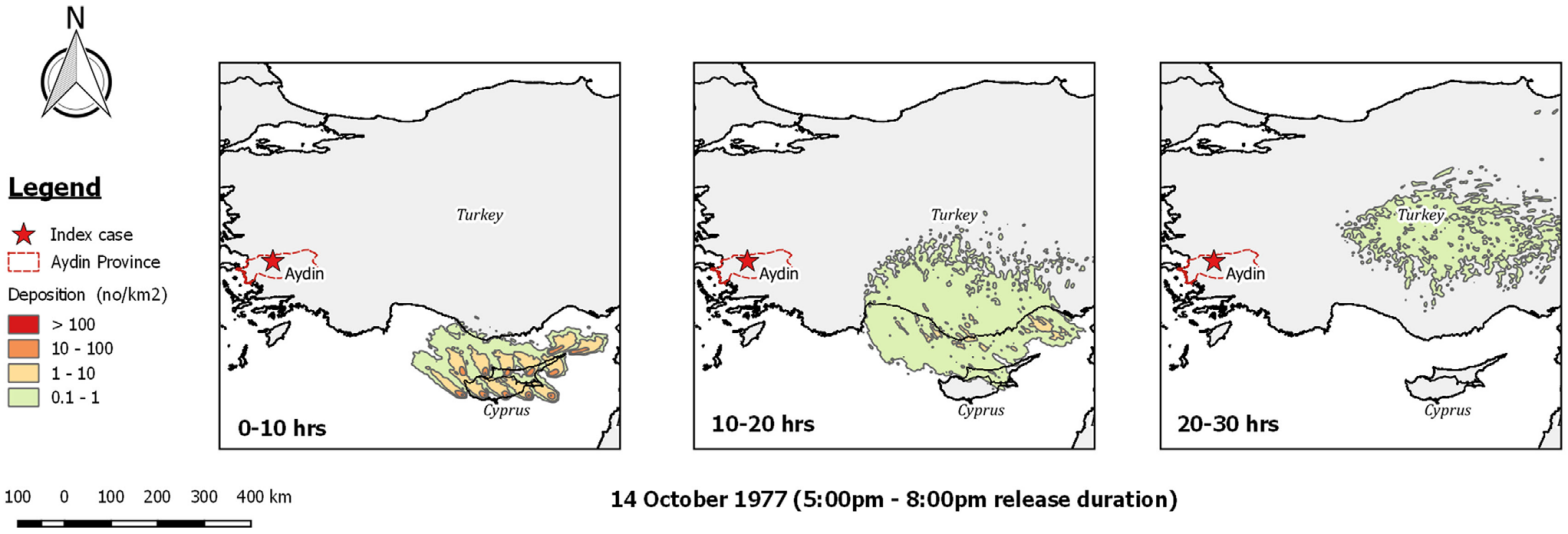
Post-processed *TAPPAS* output for a forward run for BT-TUR-77 for the evening of the 14th October 1977 indicating that even with a 30-h transport time, infected *Culicoides* midges from Cyprus could not have been the direct source of the bluetongue outbreak reported in villages near the city of Aydin on the 24th October 1977.

Sellers investigated this possibility and concluded that wind conditions on the evening of the 14th of October were suitable for such transport to occur (14). However, our reanalysis of dispersion from Cyprus using *TAPPAS* shows that while spread to the south coast of Turkey was possible, the direction of the wind was misplaced for infected midges to reach western Turkey within the upper limit currently considered for *Culicoides* survival, i.e., 30 h (Figure 7). Similarly, undertaking TAPPAS runs for the preceding 4 weeks did not find a single morning or evening when LDWD from Cyprus to western Turkey was possible.

**Figure 8:**
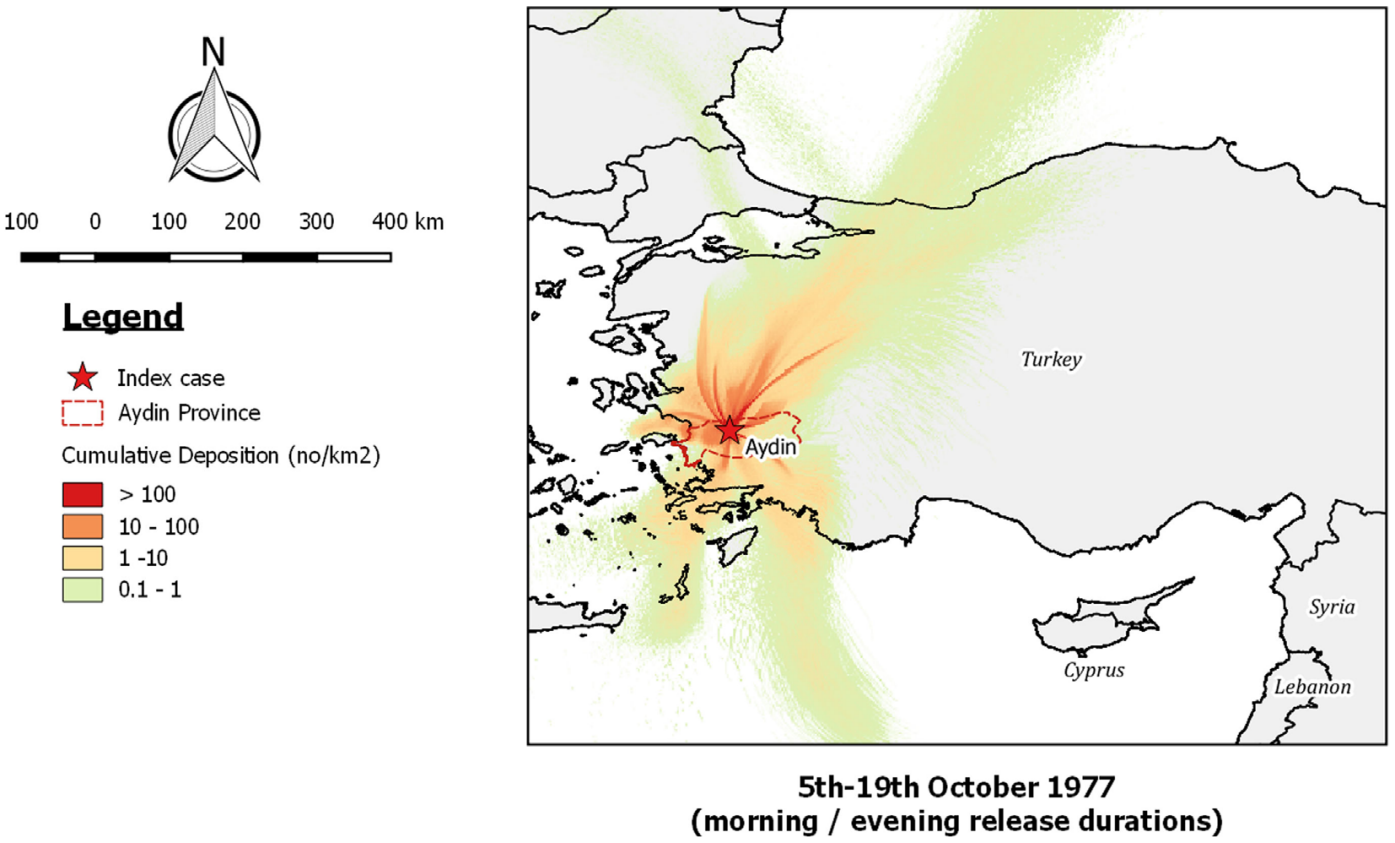
Post-processed output of a backward *TAPPAS* run for 30 hours for the outbreak of bluetongue in Aydin (BT-TUR-77) for the deposition window identified by Sellers (5th–19th October 1977). These results agree with those of the forward dispersion run (Figure 7) and thus provide additional evidence that long-distance wind dispersion from Cyprus was not a possible source of the Aydin outbreak.

Running *TAPPAS* in backward mode allowed us to determine potential source locations for the Aydin outbreak during the infection window defined by Sellers (5th–19th October, 1977). This showed that if LDWD was the cause of the outbreak it would have needed to be from a region in western Turkey or possibly some of the islands in the Aegean Sea, and that LDWD from Cyprus was not possible (Figure 8).

### BT-USA-82

Bluetongue virus, subsequently determined to be serotype 10, was first isolated in the USA in sheep in California in 1953 (48). Subsequently, BTV-11, BTV-13, and BTV-17 were isolated between 1955 and 1967, and the virus was confirmed by a sero-survey to be widespread in most of the country (49). In Florida, BTV-13 and BTV-17 had both been detected in 1967, and to investigate the epidemiology of the virus in the state, in 1981 sentinel cattle herd surveillance was commenced in three locations: Brooksville, Ona, and Belle Glade (50). In late 1982, the monthly sampling detected seroconversion to BTV in calves kept at Ona, which further testing established to be due to a serotype 2 infection. A later study using gel-electrophoresis established that there were in fact two distinct BTV-2 viruses that had infected the cattle (“OnaA and OnaB”) detected in the September and October 1982 sampling, respectively (51). OnaA was indistinguishable from the South African reference strain, originally isolated in 1959, a result subsequently confirmed by sequencing five of the strain’s 10 segments (52).

At the time of the reporting, the source of infection was not known, but it was speculated that BTV-2 might be circulating in the Caribbean, as a survey in 1982–1983 detected serotypes (BTV-6 and BTV-14) which at the time had not been identified in the USA (53). Sellers investigated the possibility that LDWD might have been the pathway of introduction, but as the potential source location was not known, he applied the “backward trajectory” approach (15). This determined the OnaA isolate might have originated in northwestern Cuba, with a presumed uplift of infected *Culicoides* on the evening of the 18th of August, with a transport time of 20 h. By contrast, examination of the trajectories for late September 1982 showed, that LDWD of the OnaB isolate from Cuba was not possible.

**Figure 9:**
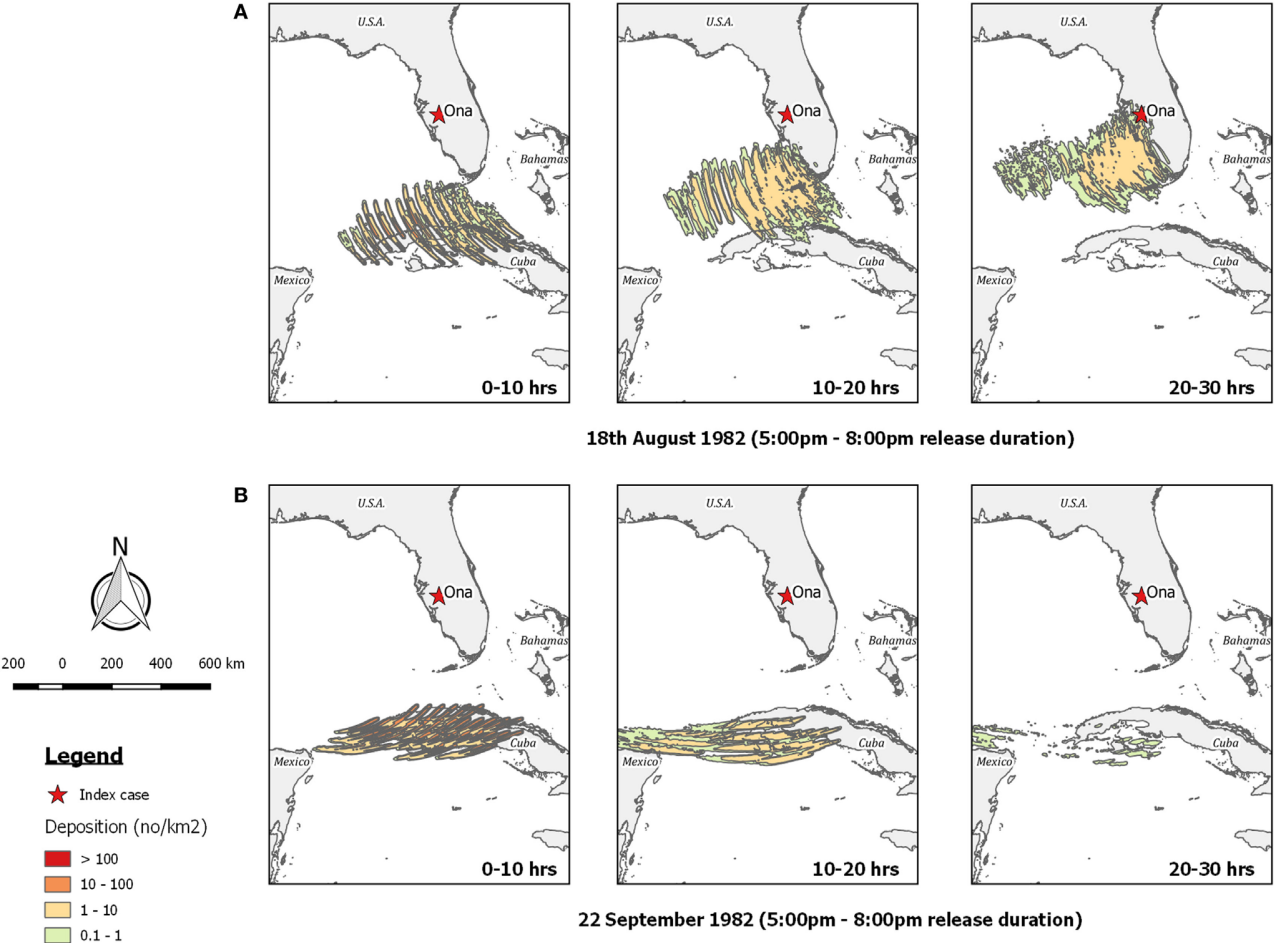
Post-processed *TAPPAS* output for BT-USA-82 showing forward dispersion from northern Cuba for three different transport times (0–10, 10–20, and 20–30 h) for **(A)** the evening of the 18th August 1982; and **(B)** the evening of the 22nd September 1982, the latter indicating long-distance wind dispersion could not have resulted in the OnaB infection.

Using *TAPPAS* for the presumed date of dispersion of OnaA we found support for the trajectory direction determined by Sellers, but showed that a flight time of 20 h was not sufficient for transporting infected midges to central Florida (Figure 9A). Extending the transport duration to 30 h made LDWD just possible, but the numbers of midges would have been low. Extending the possible LDWD time period from the 15th to the 31st August (which covers the total possible transport window, Table 2) did not show any other mornings or evenings when midges could possibly have reach central Florida.

Regarding the OnaB detection, our *TAPPAS* runs for the 21st to 23rd August agreed with Sellers’ conclusion that LDWD was not a possible incursion pathway as the winds were blowing in a westerly direction (Figure 9B).

## Discussion

When Sellers wrote the first paper suggesting that AHS might be introduced *via* wind dispersion of its midge vector (11), there was very little evidence that an arbovirus might be spread in this manner. Indeed, the supporting literature was derived almost entirely from Australia, based on observations of outbreaks of bovine ephemeral fever (BEF), caused by the BEF virus (genus: *Ephemerovirus)*. This produces an acute, but transient fever in cattle, and although the insect vector has never been definitively established, it is believed that *Culicoides* species play a role in transmission (54). Large outbreaks occurred in Australia in 1936–1937, 1955–1956, and 1967–1968 and in each of these there was a rapid spread from the Northern Territory eastwards to north Queensland and then down the east coast to as far south as the east of Victoria. As the disease is so clinically distinct, this spread could be easily tracked, and from the analysis of the reports, it was apparent that the “infection front” progressed faster than the possible movement of cattle. Thus it was suggested that wind-borne transmission of the insect vector must be main means of farm-to-farm transmission (55). Similar conclusions were reached following analyses of the 1967–1968 outbreak (56, 57).

African horse sickness was an appropriate choice made by Sellers to commence his explorations of LDWD (11) as, like BEF, it has a short incubation period, a high attack rate and obvious symptoms, and thus outbreaks can be readily detected by syndromic surveillance. Thus, for the epidemic of AHS in Morocco, the dates of the peak of the outbreak in the northernmost provinces and the subsequent spread to Spain could be more or less precisely estimated (37, 40). Accordingly, it was possible to define the spatiotemporal window of where and when infected midges might have been dispersed on wind currents. Thus it is not too surprising that Sellers’ conclusion that wind was a plausible pathway for the introduction of AHSV into Spain in 1966 was supported by our analysis using *TAPPAS* (Figure 4). This similarly applies to our conclusion supporting Sellers’ conjecture on the introduction of AHSV into Cyprus in 1960 (Figure 3).

As compared to AHS, BT is a much more complex disease with respect to its epidemiology, clinical presentation, and diagnosis, and thus it is much harder to clearly define possible spatiotemporal windows for LDWD in source countries, and to rule out other pathways, including that the virus might be already present in the country. Accordingly, of the four BT outbreaks we reanalyzed, only for one of these (BT-CYP-77) could we find some support for the LDWD for the transport window indicated. Even then, there are puzzling features of this outbreak, particularly in it initially occurring in only two specific districts of the island whereas the cumulative dispersion modeling indicates that outbreaks should have been more widely dispersed (Figure 6). As was noted by Sellers (13), Famagusta and Kyrenia were exactly the same districts where outbreaks had occurred previously, and thus they represented “hot spots” for the disease. Thus it is possible that that these districts had management or environmental risk factors which facilitated the survival of LDWD midges and/or the onward propagation of the virus within the local sheep population. This hypothesis has support from an observation made by a Cypriot veterinary official that many BT “outbreaks commence in areas of irrigation (Famagusta district) or areas fed by natural springs (Kyrenia district)” (44).

For the 1956 outbreak of BT in Portugal, Sellers had presumably neither direct knowledge of it nor access to additional, unpublished data from either Portugal or Morocco. Thus he relied entirely on the papers published about the outbreaks in the two countries (Table 2). A underlying assumption in assigning a LDWD window is that the first *detected* case is one of the first *infected* cases in the destination country. This is a reasonable assumption when there is heightened awareness of the possibility of the introduction of disease—as occurred subsequently with AHS in Cyprus in 1960 and Spain in 1966—but without this augmented surveillance, then this assumption may not be valid, given current understanding of farmer reporting of unexpected exotic animal disease (58). Thus, it is not an entirely unrealistic scenario, assuming that LDWD of infected *Culicoides* occurred on the earlier date suggested by our modeling, that the disease was not reported for another 7 weeks, upon movement of sheep from Alcacer do Sal onto the farm where it was detected. On the other hand, the 1956 outbreak did cause marked lesions in the affected sheep (31), and thus it can also be equally argued that it is unlikely that the disease would have gone unrecognized for so many weeks, and thus LDWD was not the true route of incursion.

The second of Sellers’ analyses not supported by our modeling is the detection of BTV-2 in the USA in September 1982, but unlike for BT-PRT-56, runs of *TAPPAS* in the previous 2 months did not detect a dispersion event of less than 20 h in which infected midges might have been carried from the north coast of Cuba to central Florida. This fact, combined with the lack of support for LDWD for the second detection (“OnaB”) on the 8th of October 1982 argues for alternative routes of introduction. The most plausible of these, which was mentioned in the original report (50), is *via* importation of a BTV-infected ruminant species from Africa. This is indicated by the molecular analysis of the OnaA isolate which showed it to be closely related to South African BTV-2 strains (52), and a serological study of antelopes imported into US zoological parks which found many had antibodies to a diversity of BTV serotypes, including BTV-2 (59). Relevant to these reports indicating the potential role of imported antelopes from Africa in introducing BTV-2 into the USA, there is a zoological park at Tampa, only approximately 60 km away from Ona. Against this hypothesis, Sellers cites a personal communication from the lead researcher of the original BTV-2 detection (E. P. J. Gibbs) that his subsequent investigations had ruled out an introduction of BTV from Africa.

Thus, it is hard to make a definitive conclusion as to what was the true introduction pathway. If it was LDWD from northern Cuba, much depends on how long midges can actually survive in the air column, and it needs to be noted that the upper limits for the transport survival time of *Culicoides* does not have any real experimental basis, and is in part based on the Sellers’ wind dispersion studies (27). There is a pressing need for laboratory studies to confirm the 20 h threshold, as well as to quantify how it might be conditional on the temperature and humidity conditions experienced by the midges during their air-column transport.

### *TAPPAS*, Big Data, and Verified Risk Assessment Software

In total, the *TAPPAS* system currently has 3.7 TB of stored meteorological data, and combined with the use of cloud computing to rapidly process multiple requested runs and quickly return to the user high quality visuals, it can be considered a “Big Data” application. The definition of what exactly defines Big Data is contentious, but the consensus is that it constitutes IT systems for rapidly processing and integrating large volumes of data which are informative to end-users for decision making. These criteria are generally summarized as the three “v” terms of Big Data: *volume* (amount of data), *velocity* (speed of data processing) and *variety* (different data types) (60). Since this conceptualization, other “v” terms have been added: *veracity* (indicating that error trapping processes can remove erroneous data), *validity* (such that the processes are replicable and follow quality standards of data management and processing) and *value* (emphasizing that the Big Data system needs to be actually useful).

Although the term “Big Data” was not used as such, one of the first widely publicized examples of its concepts in public health was *Google Flu Trends*, which used internet search terms to predict an upsurge in seasonal influenza in the USA 7–10 days before the system in place by the CDC relying on physician reports (61). This data mining of the Internet for syndromic (clinical) surveillance can now be recognized as one of the main potential Big Data approaches, and although not as common as in public health, examples of its use in animal health are now starting to appear. For example, Guernier *et al*. (62) describe the use of *Google Trends* to monitor the occurrence of tick paralysis in companion animals in eastern Australia, potentially facilitating the early detection of high-risk periods of the disease.

Many of the earlier references to the use of Big Data in the biomedical literature made reference to its potential for genomic data and associated bioinformatics analyses, particularly with respect to data storage and processing using cloud computing (63). This applies equally to veterinary science as to other disciplines, and Kao *et al*. (64) describe the specific approach of integrating whole-genome sequences with animal movement data to better inform infection networks.

**Figure 10:**
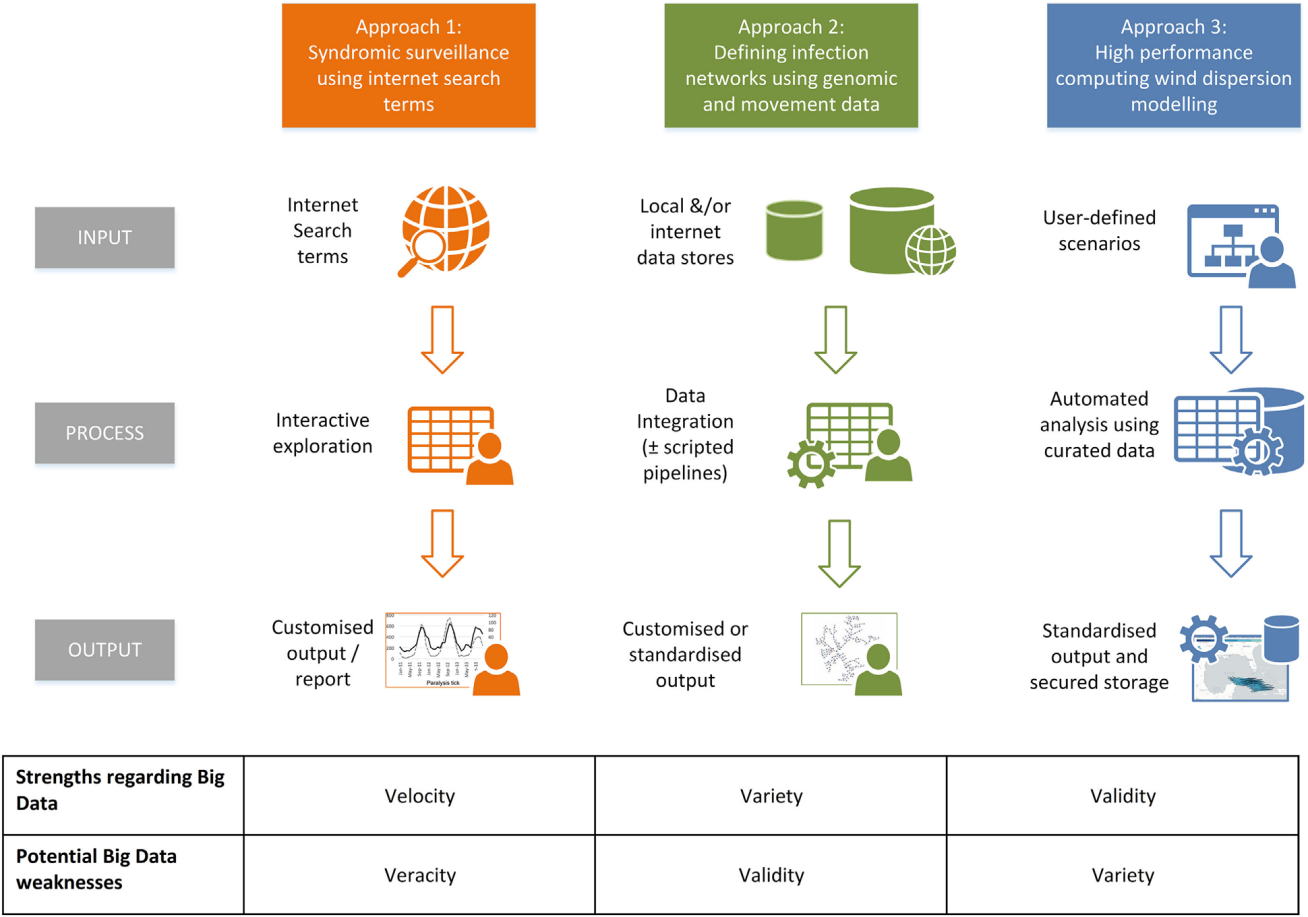
An infographic summarizing examples of Big Data approaches reported in the veterinary literature: approach 1, data mining of internet terms to determine temporal trends in disease syndromes (62); approach 2, the integration of publically accessible genomic with animal movement data to define the “paths of transmission” of infectious diseases (64); and approach 3, dispersion modeling of curated atmospheric data with long-term storage of output to ensure the results are replicable (22), of which *TAPPAS* is also an example.

Generalizing these Big Data approaches in terms of processes, it can be seen that each are tacking different aspects of the six potential “v” terms (Figure 10). Thus the data-mining approach using Internet search terms highlights the potential for “velocity” (in rapidly being able to detect emerging trends), but this can be at the cost of veracity, particularly with respect to “false alarms” (65). Considering the development of infection network systems which integrate genomic and contact data, issues can arise with the complexity of the analyses, with the need to choose between competing and rapidly developing algorithms, each possibly giving different predictions (66). Regarding *TAPPAS* [and comparable systems, e.g., Ref. (22)] as examples of Big Data approaches, their strength lies in the focus on validity, in only using high quality, curated meteorological datasets, and with having all the analyses steps automated. Furthermore, the output of the runs is stored, making any use of it for scientific inquiry truly replicable. However, such system are relatively expensive and time-consuming to build, and *TAPPAS* at least, currently only handles the dispersion part of the total risk pathway, and cannot assess in a rigorous way “uplift” at the source (i.e., are there actually *Culicoides* midge populations there?) or “propagation” at the destination (i.e., will the dispersed midges that survive transport be able to onwardly transmit viruses?). To answer these questions requires the addition of a greater “variety” of datasets and algorithms.

## Conclusion

When Sellers wrote his papers between 1977 and 1992 suggesting that long-distance dispersal of *Culicoides* midges by wind might be a mechanism for the spread of BTV and AHSV, there was at the time no experimental support, and the inference was based purely on “ruling out” other pathways, especially the movement of animals. Combining this with retrospective observations of wind direction and meteorological conditions, he was able to conclude that wind-dispersed infected *Culicoides* was a possible source of introduction for most of his case studies.

In the interim period, there has been a steady accumulation of experimental evidence and field observations to support LDWD of *Culicoides* as a plausible pathway of introduction of BTV and AHSV. The most important documented case studies are from the outbreaks of BTV-8 in England and Sweden, where the heightened surveillance at the time means that any other possible pathway can be excluded (22). However, in the endemically infected BTV/AHSV countries, where both midges and viruses may be present at both the source and the destination, the situation is more complex, and dispersion modeling taken by itself needs to be used cautiously. Where it is of undoubted value is, as in the case of BT-TUR-77, in enabling the “ruling out” of LDWD as a possible means of spread. However, to properly “rule in” LDWD requires additional data and modeling, of which the use of genomic data of both the vector midge and the viruses is playing an increasingly important role (67–71). We predict this will be particularly useful in the situation where the outbreak location and the purported origins are connected by roads within the same landmass, and thus the movement of animals and products is more likely than over the sea (72). But irrespective of whether the LDWD is over the sea or land, the challenge is to develop validated systems which integrate LDWD modeling with infection network reconstruction. We suggest that this can be assisted by borrowing insights and techniques from Big Data science where the competing objectives of managing data quantity while maintaining data quality are fully recognized and solutions are actively being developed.

## Author Contributions

PD conceived the study, designed the *TAPPAS* software, analyzed the results, and wrote the first draft of the paper. KG oversaw the development of the *TAPPAS* software, analyzed the results, and edited the final draft of the paper. RK managed the *TAPPAS* project and edited the final draft of the paper.

## Conflict of Interest Statement

The authors declare that the research was conducted in the absence of any commercial or financial relationships that could be construed as a potential conflict of interest.

## Footnotes

^1^https://research.csiro.au/tappas/.

^2^https://openlayers.org/.

## Abbreviations

ADM, atmospheric dispersion models; AGL, above ground level; AHS, African horse sickness; AHSV, African horse sickness virus; BT, bluetongue; BTV, bluetongue virus; *HYSPLIT*, Hybrid Single-Particle Lagrangian Integrated Trajectory; LDWD, long-distance wind dispersion; NWP, numerical weather prediction; OIE, World Organisation for Animal Health; *TAPPAS*, Tool for Assessing Pest and Pathogen Airborne Spread.
